# Custodiol-N, the novel cardioplegic solution reduces ischemia/reperfusion injury after cardiopulmonary bypass

**DOI:** 10.1186/s13019-015-0226-9

**Published:** 2015-02-28

**Authors:** Gábor Veres, Tamás Radovits, Béla Merkely, Matthias Karck, Gábor Szabó

**Affiliations:** 1 Department of Cardiac Surgery, University of Heidelberg, Heidelberg, Germany; 2 Heart and Vascular Center, Semmelweis University, Budapest, Hungary

**Keywords:** Cardiopulmonary bypass, Myocardial protection, HTK, Cardioplegic solution, Custodiol, Ischemia-reperfusion injury

## Abstract

**Backgrounds:**

On the basis of Custodiol preservation and cardioplegic solution a novel cardioplegic solution was developed to improve the postischemic cardiac and endothelial function. In this study, we investigated whether its reduced cytotoxicity and its ability to reduce reactive oxygen species generation during hypoxic condition have beneficial effects in a clinically relevant canine model of CPB.

**Methods:**

12 dogs underwent cardiopulmonary bypass with 60 minutes of hypothermic cardiac arrest. Dogs were divided into 2 groups: Custodiol (n = 6) and Custodiol-N (n = 6) (addition of L-arginin, N-α-acetyl-L-histidine and iron-chelators: deferoxamine and LK-614). Left ventricular hemodynamic variables were measured by a combined pressure-volume conductance catheter at baseline and after 60 minutes of reperfusion. Coronary blood flow, myocardial ATP content, plasma nitrate/nitrite and plasma myeloperoxidase levels were also determined.

**Results:**

The use of Custodiol-N cardioplegic solution improved coronary blood flow (58 ± 7 ml/min vs. 26 ± 3 ml/min) and effectively prevented cardiac dysfunction after cardiac arrest. In addition, the myocardial ATP content (12,8 ± 1,0 μmol/g dry weight vs. 9,5 ± 1,5 μmol/g dry weight) and plasma nitrite (1,1 ± 0,3 ng/ml vs. 0,5 ± 0,2 ng/ml) were significantly higher after application of the new cardioplegic solution. Furthermore, plasma myeloperoxidase level (3,4 ± 0,4 ng/ml vs. 4,3 ± 2,2 ng/ml) significantly decreased in Custodiol-N group.

**Conclusions:**

The new HTK cardioplegic solution (Custodiol-N) improved myocardial and endothelial function after cardiopulmonary bypass with hypothermic cardiac arrest. The observed protective effects imply that the Custodiol-N could be the next generation cardioplegic solution in the protection against ischemia-reperfusion injury in cardiac surgery.

## Background

The development of cardioplegic solutions was one of the major advances in cardiac surgery that allowed surgeons to extend period of ischemic arrest over 3 hours to perform complex surgical procedures without adversely affecting myocardial function. Among cardioplegic solutions, Custodiol is unique because it could be used for myocardial protection in complex cardiac surgery and for organ transplantation in transplant surgery. Furthermore, Custodiol is attractive for longer cardiac surgery procedures, because it is administered as a single dose and is proved to offer myocardial protection for a period of up to four hours [1,2]. Robinson et al. [3] examined the method of myocardial protection used by North-American surgeons and they showed that 98% of the surgeons used cardioplegic arrest and that 72% of them used blood cardioplegia, 22% crystalloid cardioplegia. In the past, several clinical studies compared the effects of Custodiol and blood cardioplegia on myocardial injury and none of them demonstrated better cardiac or endothelial function [4]. Nevertheless, a recent study demonstrated that one single dose of antegrade Custodiol is as effective as repetitive antegrade cold blood cardioplegia in protecting the myocardium [5]. Interestingly, Scrascia et al. [6] found that Custodiol provided an improved cardiac protection in longer ischemic times when compared to blood cardioplegia.

Recent patient demographic changes, with surgeons operating on older and sicker patients who have more severe disease, potentially requires a more prolonged ischemia, hence, an improved myocardial and endothelial protection would be of benefit.

Although, hypothermic cardiac arrest is a widely accepted protective concept, recently, it has been shown that cold temperature and ischemia/ reperfusion may lead to definitive myocyte and endothelial injury mainly mediated by an iron-dependent formation of reactive oxygen species (ROS). Based on novel insights in cold-induced cell and tissue injury [7,8], we have recently developed an improved new modification of the former Custodiol solution, which proved to be of superior quality in cell culture experiments as well as preservation of liver and lung [7,9-12]. In our previous studies in small animals [13,14], several Custodiol-based cardioplegic solutions were also tested in vitro as well as in vivo to obtain the ideal composition of a new cardioplegic solution. In our previous investigations, we demonstrated the best cardioprotective effect of Custodiol-N (composition of Custodiol-N see Table 1.) among the new Cusodiol-based cardioplegic solutions against ischemia/reperfusion injury [13,14].

**Table 1:** **Compounds of evaluated cardioplegic solutions**

Compounds of evaluated cardioplegic solutions		
	Custodiol mmol/L	Custodiol-N mmol/L
Na^+^	16	16
K^+^	10	10
Mg	4	8
Ca^2+^	0.015	0.020
CI	50	30
L-histidine	198	124
N-α-acetyl-L-histidine	-	57
Tryptophan	2	2
a-ketoglutarate	1	2
Aspartate	-	5
Arginine	-	3
Alanine	-	5
Glycine	-	10
Mannitol	30	-
Sucrose	-	33
Deferoxamine	-	0.025
LK-614	-	0.0075

Composition of the clinically used Custodiol and Custodiol-N solution.

Based on the reassuring results of the aforementioned studies we planned a preclinical, experimental study to examine the efficacy and the safety of the newly developed cardioplegic solution (Custodiol-N) compared to the Custodiol solution on cardiac and endothelial function in a clinically relevant canine model of cardiopulmonary bypass.

## Methods

### Animals and experimental groups

Twelve dogs (foxhounds) weighing 21 to 34 kg (25.5 ± 1.4 kg) were used in this experiment. All animals received humane care in compliance with the Principles of Laboratory Animal Care formulated by the National Society for Medical Research and the Guide for the Care and Use of Laboratory Animals prepared by the Institute of Laboratory Animal Resources and published by the National Institutes of Health (NIH Publication No. 86–23, revised 1996). The experiments were approved by the Ethical Committee of the Land Baden-Württemberg for Animal Experimentation. After the dogs were randomly assigned to the experimental groups, dogs were divided into 2 groups: Custodiol (n = 6) and Custodiol-N (n = 6).

### General management and cardiopulmonary bypass

The dogs were premedicated with propionylpromazine and anesthetized with pentobarbital (15 mg/kg initial bolus and then 0.5 mg/kg/h i.v.), paralyzed with pancuronium bromide (0.1 mg/kg as a bolus and then 0.2 mg/kg/h i.v.), and endotracheally intubated. The dogs were ventilated with a mixture of room air and O_2_ (FiO_2_ = 60%) at a frequency of 12–15/min and a tidal volume starting at 15 ml/kg/min. The settings were adjusted to maintain arterial partial carbon dioxide pressure levels between 35 and 40 mmHg. The femoral artery and vein were cannulated to record aortic pressure (AoP) and to take blood samples for the analysis of blood gases, electrolytes, pH and further biochemical measurements (see below). Basic intravenous volume substitution was carried out with Ringer solution (1 ml/min/kg). According to the values of potassium, bicarbonate, and base excess, substitution included administration of potassium chloride and sodium bicarbonate (8.4%). Neither catecholamines nor other hormonal or pressor substances were administered. After left anterolateral thoracotomy in the fourth intercostal space and pericardiotomy, the great vessels were dissected. After systemic anticoagulation with sodium heparin (300 U/kg), the left subclavian artery was cannulated for arterial perfusion. The venous cannula was placed in the right atrium. The extracorporeal circuit consisted of a heat exchanger, a venous reservoir, a roller pump, and a membrane oxygenator primed with Ringer lactate solution (1000 ml) supplemented with heparin (150 U/kg) and 20 ml sodium bicarbonate (8.4%). After initiation of CPB, the body temperature was cooled to 28°C. After crossclamping of the aorta, the heart was arrested with different cardioplegic solutions based on the actual protocol (Custodiol, Custodiol-N). During cardiac arrest the pump flow was set at 100 ml/kg/min to maintain perfusion pressure above a value of 35–40 mmHg at any time point, and alpha-stat management was applied. Twenty minutes before cross-clamp removal, rewarming was initiated. After 60 min of cardiac arrest, the aorta was declamped, and the heart was reperfused with normothermic blood in the bypass circuit. If necessary, ventricular fibrillation was counteracted with cardioversion of 40 J. Ventilation was restarted with 100% oxygen. After a total time of 90 minutes the animals were weaned from CPB. All animals were weaned from CPB without inotropic support 20 min after the release of the aortic cross clamp. Each animal of the CPB groups underwent 90 min of CPB with 60 min of cardiac arrest and 60 min of reperfusion.

### Measurements of cardiac and coronary vascular function in vivo

Left ventricular systolic and diastolic pressures and volumes were measured by a combined 6 F Millar pressure-volume conductance catheter with 6 mm spacing, which was inserted via the apex. Stroke volume (SV) was calculated from the integrated flow signal measured by an aortic ultrasonic flow probe (Transonic Systems Inc., Ithaca, NY, USA) and was used to calibrate the volume signal from the conductance catheter. Parallel conductance was estimated by rapid injection of 1 ml of hypertonic saline into the left atrium. The pressure and volume signal provided by the conductance catheter was registered continuously and computed by the IOX software (EMKA Technologies, Paris, France). Vena cava occlusions were performed to obtain a series of pressure–volume loops. The slope (E_es_) of the left ventricular end-systolic pressure–volume relationship and preload recruitable stroke work (PRSW) were calculated as load-independent indices of myocardial contractility.

Coronary blood flow was measured on the left anterior descending (LAD) coronary artery with a perivascular ultrasonic flow probe (Transonic Systems Inc., Ithaca, NY, USA). Endothelium-dependent coronary vasodilatation was assessed after intracoronary administration of a single bolus of acetylcholine (ACh, 10^−7^ mol) and endothelium-independent vasodilatation after administration of sodium-nitroprusside (SNP, 10^−4^ mol). The vasoresponse was expressed as percentage change of CBF. At the end of the experiments left ventricular myocardial tissue samples were taken and immediately immersed in fluid nitrogen (−196°C) and stored frozen at −80°C until the biochemical measurements.

### Biochemical measurements

For determination of the myocardial energetic state myocardial adenosine triphosphate (ATP), adenosine diphosphate (ADP), and adenosine monophosphate (AMP) contents were assessed from myocardial tissue with standard photometry using an enzyme-kinetic assay.

Plasma nitrate/nitrite levels (as an index of in vivo total NO-production) were determined by a colorimetric assay according to the Griess-method using a commercial kit (Cayman Chemical Co., Ann Arbor, MI, USA).

Plasma myeloperoxidase level (as a marker for systemic inflammation and leukocyte activation) was measured by enzyme linked immunosorbent assay (ELISA) using a commercial kit (EMD Biosciences Inc. La Jolla CA, USA).

### Drugs

Acetylcholine (ACh) and sodium nitroprusside (SNP) were from Sigma-Aldrich, Germany. Custodiol and Custodiol-N were obtained from Dr. Franz Köhler Chemie GmBH, Alsbach-Hähnlein, Germany.

### Statistical analysis

All measurements were performed before cardiopulmonary bypass and after 60 min of reperfusion. All values were expressed as mean ± standard error of the mean (SEM) or standard deviation (SD). A paired *t*-test was used to compare two means within a group (comparison of “before” and “after” values). Means between the groups were compared by an unpaired two-sided Student’s *t*-test (comparison of Custodiol and Custodiol-N groups). A probability value less than 0.05 was considered statistically significant. In the figures only the significances between the groups were indicated.

## Results

### Haemodynamics

Heart rate (HR), mean arterial pressure (MAP), cardiac output (CO) and coronary blood flow (CBF) are shown in Table 2. Baseline heart rate was identical in the groups. MAP showed decreased values in both groups after CPB. CO and CBF were comparable in both groups at baseline and CO remained unchanged in both groups after CPB. After 60 min of reperfusion, CBF was significantly higher in the Custodiol-N group when compared to Custodiol.

**Table 2:** **Haemodynamic and biochemical parameters**

	Before CBP		After CPB	
	Custodiol	Custodiol-N	Custodiol	Custodiol-N
HR (1/min)	122.5 ± 8.1	144.3 ± 8.4	130.1 ± 4.6	157.5 ± 13.1
MAP (mmHg)	89 ± 3	79 ± 5	77 ± 11	67 ± 3
CO (l/min)	2.1 ± 3.9	2.3 ± 0.4	1.9 ± 0.2	2.3 ± 0.2
CBF (ml/min)	41.3 ± 6.1	44.5 ± 3.8	26.2 ± 2.8#	58 ± 7.0^*^
MPO (ng/ml)	0.9 ± 0.3	1.1 ± 0.1	4.3 ± 2.2#	3.4 ± 0.4#^*^
Nitrite (ng/ml)	1.2 ± 0.3	1.6 ± 0.3	0.5 ± 0.2#	1.1 ± 0.3^*^

HR: heart rate, MAP: mean arterial pressure, CO: cardiac output, CBF: coronary blood flow, MPO: plasma myeloperoxidase. All values are given as mean ± SEM. *: p < 0.05 vs. Custodiol, #: p < 0.05 vs. baseline.

### Left ventricular function

**Figure 1:**
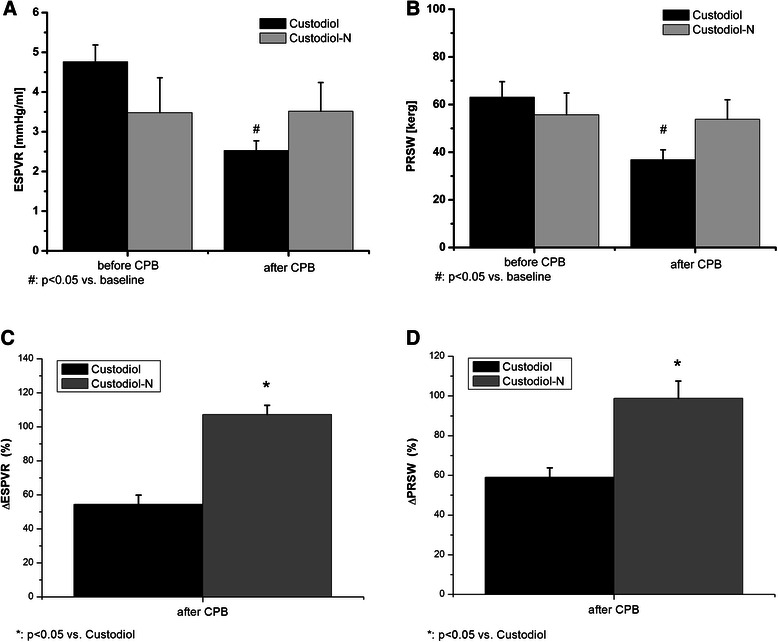
**Changes of the slope of (A) left ventricular end-systolic pressure volume relationship (ESPVR) and (B) left ventricular preload recruitable stroke work (PRSW) before and after cardiopulmonary bypass and 60 minutes of reperfusion.** Relative changes of the slope of **(C)** left ventricular end-systolic pressure volume relationship (ESPVR) and **(D)** left ventricular preload recruitable stroke work (PRSW) after cardiopulmonary bypass and 60 minutes of reperfusion. All values are given as mean ± SEM. *p < 0.05 vs. Custodiol, #p < 0.05 vs. baseline.

Left ventricular function was similar in both groups at baseline (Figure 1). Myocardial contractility - characterized by the load-independent indexes Ees (slope of end-systolic pressure-volume relationship (ESPVR) and preload recruitable stroke work (PRSW) (Figure 1) -showed a significant decrease (p < 0.05) after extracorporal circulation and reperfusion in the Custodiol group while it remained unchanged in the Custodiol-N group.

### Endothelial function in vivo

**Figure 2:**
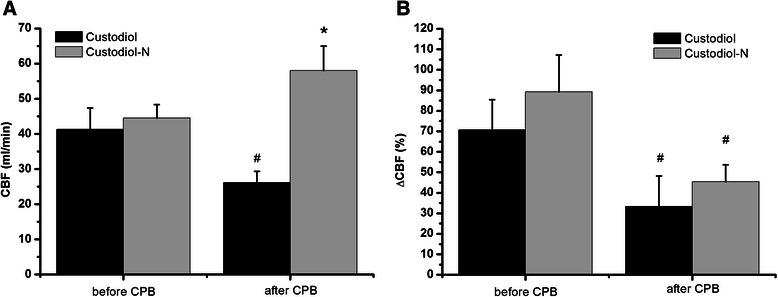
**Coronary vascular function.** Baseline coronary blood flow **(A)** and **(B)** endothelium-dependent vasodilatation after acetylcholine (ACh, 10^−7^ mol) expressed as a percent change of baseline coronary blood flow. All values are given as mean ± SEM. *p < 0.05 vs. Custodiol, #p < 0.05 vs. baseline.

Coronary blood flow was similar in both groups before cardioplegic arrest. After 60 min of reperfusion, coronary blood flow decreased significantly (*p* < 0.05) in the Custodiol group, but it increased in the Custodiol-N group (Table 2. Figure 2A). Endothelium-dependent vasodilatation after ACh was significantly (p < 0.05) reduced both groups after 60 min of reperfusion in comparison to values before extracorporal circulation (Figure 2B). Endothelium-independent vasodilatation after SNP showed no significant differences over time or between groups (data not shown).

### Biochemical measurements

Plasma nitrate/nitrite and myeloperoxidase values did not significantly differ between the groups at baseline. However, myeloperoxidase level was significantly higher in the traditional HTK group than in the Custodiol-N group (Table 2). Furthermore, there was also lower level of plasma nitrate/nitrite in the Custodiol group in comparison to the treatment group (Custodiol-N) after CPB (Table 2).

Myocardial high energy phosphate content is listed in Table 3. ATP values were significantly higher in the Custodiol-N group after 60-min reperfusion than in the Custodiol group (Table 3).

**Table 3:** **Myocardial high energy phophates**

	Custodiol	Custodiol-N
ATP (μmol/g dry weight)	9.5 ± 1.5	12.8 ± 1.0*
ADP (μmol/g dry weight)	5.6 ± 1.0	5.6 ± 0.5
AMP (μmol/g dry weight)	1.5 ± 0.3	1.7 ± 0.3

Myocardial levels of adenosine triphosphate (ATP), adenosine diphosphate (ADP) and adenosine monophosphate (AMP) are shown after cardiopulmonary bypass in both groups. *: p < 0.05 vs. Custodiol.

## Discussion

We demonstrated the superiority of Custodiol-N above Custodiol in terms of cardiac and endothelial function after CPB in our clinically relevant canine model of cardiopulmonary bypass.

Since the start of cardiac surgery in the 1950s, multiple techniques (hypothermia, crystalloid or blood cardioplegic solutions, direction of introduction (antegrade or retrograde) etc.) have been used to protect the heart during the surgical requirement for elective global ischemia. However, no single method was unequivocally the best. There is a clinical and experimental evidence of IR injury after CPB. Even if cardiac dysfunction is not always clinically remarkable after CPB, reduction of myocardial contractility may occur as described in a human study using pressure–volume relationships [15]. We could also show in our previous canine studies, that myocardial dysfunction was developed after the administration of Custodiol using a sensitive pressure-volume conductance catheter [16].

The new cardioplegic solution (Custodiol-N) was modified in 4 important points to enhance the protective capacity of the Custodiol solution: 1, the addition of iron-chelators (desferoxamine and the new, membrane-permeable LK-614) to reduce iron-dependent injury 2, reduction of chloride concentration to reduce chloride-induced injury 3, addition of cardioprotective amino acids (L-Arginin: NO-precursor to improve coronary blood flow; glycine and alanine to stabilize the plasma membrane) 4, partially substitution of histidine by N-α-acetyl-L-histidine to inhibit the histidine-induced cytotoxicity.

The combination of hypothermia and the potassium-induced cardiac arrest represent the basis concept of cold crystalloid cardioplegic solutions. However, present studies clearly demonstrated that cold storage activates specific cardiac injury processes; production of ROS triggered by a cold-induced rise of physiologically negligible cellular pool of the cellular cheletable iron, which inhibit strongly cardiac and endothelial function since redox-active iron ions convert ROS of low toxicity into highly reactive species such as hydroxyl radicals [17,18]. Rauen and co-workers determined the chelatable iron pool in cultured rat hepatocytes and showed that at low temperature the chelatable iron pool rapidly increased and remained elevated for some hours [19]. In a further investigation, Rauen et al. demonstrated the effectiveness of a non-permeable iron-chelator deferoxamine and a new membrane permeable iron-chelator LK-614 against iron-dependent cold injury [8].

Recently, a new, iron-independent component of cold induced injury has also been described. When the stronger iron-dependent component was inhibited with iron chelators, the previously overshadowed chloride-dependent injury became the limiting factor of cell survival. The new solution (Custodiol-N) contains only 30 mM of chloride because this concentration was sufficient to inhibit the iron-independent component of cold induced injury in cultured rat hepatocyte; there was no need to eliminate chloride completely from the medium [7].

An experimental study clearly showed that application of L-arginine as a substrate of eNOS can improve postischemic blood flow and decrease leukocyte adhesion [20]. Moreover, a recent human study described that application of L-arginin into the cardioplegic solution improved left ventricular diastolic function [21]. Two amino acids, alanine and glycine, are also included in Custodiol-N in order to decrease the sodium accumulation during cold ischemia and to stabilize the plasma membrane [12].

Side effects of using Custodiol solution were previously also described (8). The main component of Custodiol, histidine, is an excellent physiological buffer. However, Rauen et al. demonstrated that histidine has a potential negative effect on cell survival during cold ischemia (called as preservation solution toxicity) [8]. With the use of iron chelators (deferoxamine and LK-614), histidine toxicity was strongly inhibited [8,13,22]. They also found that a histidine-derivative, N-acetylhistidine exerted almost no toxicity [8,13]. To prevent the injury caused by histidine, N-acetylhistidine was added into the new Custodiol-N. Radovits et al. [13] reported in an in vitro experiment a significantly better endothelium protection with an N-acetylhistidine containing solution (without any additives) when compared to Custodiol.

The new cardioplegic solution (Custodiol-N) has already been showed largely improved liver, lung and heart preservation in different experimental studies [9-11,14]. Based on the results of these studies we have tested the effect of Custodiol-N on cardiac and endothelial function in a clinically relevant large animal model. In this study, we investigated global myocardial performance and endothelial function and metabolic parameters of the heart after a 60 minutes cardioplegic arrest. The clinically remarkable parameters such as CO and MAP were unchanged after CPB in both groups. However, we could demonstrate that Custodiol-N is more effective than Custodiol at improving the sensitive, load-independent index of left ventricular contractility (ESPVR, PRSW) after hypothermic cardioplegic arrest and reperfusion. In particular, the PRSW of the left ventricle in the Custodiol-N group remained practically unchanged when compared with the baseline to 60-minute reperfusion values (Figure 1).

To prove whether the Custodiol-N reduce ROS-generation, we also measured biochemical parameter of heart such as plasma myeloperoxidase and plasma nitrite/nitrate level. The increased level of nitrite may be explained as a result of better cardioprotection. Plasma nitrite in blood has been widely used as an index of endothelial NO synthase activity as routine indirect measures of NO levels [23]. Myeloperoxidase (MPO) is mainly released by neutrophil granulocytes, characterised by powerful pro-oxidative properties [24]. MPO catalyzes the conversion of chloride and hydrogen peroxide to hypochlorite and is secreted during inflammatory response. As hypochlorite is extremely toxic to mammalian cells, it causes tissue degradation and DNA breakage. In addition, MPO consumes endothelial-derived NO, thereby reducing NO bioavailability and impairing its vasodilating and anti-inflammatory properties. As shown by the metabolic values, application of new iron-chelators and N-acetyl-L-histidine into Custodiol-N seems to represent an optimal cardioprotection in compared to Custodiol, because hearts of this group showed the best recovery of energy charge potential and cardiac performance. In addition, the increased ATP level in the Custodiol-N group is also a sign of a better cardioprotection.

Cardioplegic solutions were originally designed to protect the myocardium, however at that time the protection of the vasculature, and more importantly, the endothelium, was not an objective. Recently, Radovits et al. [13] demonstrated the inability of Custodiol solution to offer a reasonable protection and preservation of endothelial function after long-term ischemic storage. Despite the importance of coronary endothelium few investigators examined the effect of Custodiol on endothelial function [25]. In the present study we measured in vivo coronary blood flow before and after 60-min reperfusion. After 60-min reperfusion the basal CBF remained unchanged in the Custodiol-N group while in the Custodiol group decreased significantly when compared to the baseline level (Figure 2).

## Conclusions

In conclusion, our large animal study provides evidence that the new, L-arginin and N-α-acetyl-L-histidine-enriched, chloride-poor, iron-chelator-enhanced cardioplegic solution (Custodiol-N) allows superior cardiac and endothelial protection compared to Custodiol after hypothermic cardiac arrest. The observed protective effects imply that the Custodiol-N could be the next generation cardioplegic solution in the protection against ischemia-reperfusion injury in cardiac surgery.
